# Modulation of firing and synaptic transmission of serotonergic neurons by intrinsic G protein-coupled receptors and ion channels

**DOI:** 10.3389/fnint.2013.00040

**Published:** 2013-05-23

**Authors:** Takashi Maejima, Olivia A. Masseck, Melanie D. Mark, Stefan Herlitze

**Affiliations:** Department of Zoology and Neurobiology, Ruhr-University BochumBochum, Germany

**Keywords:** 5-HT system, GPCRs, auto-regulation, hetero-regulation, optogenetics

## Abstract

Serotonergic neurons project to virtually all regions of the central nervous system and are consequently involved in many critical physiological functions such as mood, sexual behavior, feeding, sleep/wake cycle, memory, cognition, blood pressure regulation, breathing, and reproductive success. Therefore, serotonin release and serotonergic neuronal activity have to be precisely controlled and modulated by interacting brain circuits to adapt to specific emotional and environmental states. We will review the current knowledge about G protein-coupled receptors and ion channels involved in the regulation of serotonergic system, how their regulation is modulating the intrinsic activity of serotonergic neurons and its transmitter release and will discuss the latest methods for controlling the modulation of serotonin release and intracellular signaling in serotonergic neurons *in vitro* and *in vivo*.

## INTRODUCTION

**FIGURE 1:**
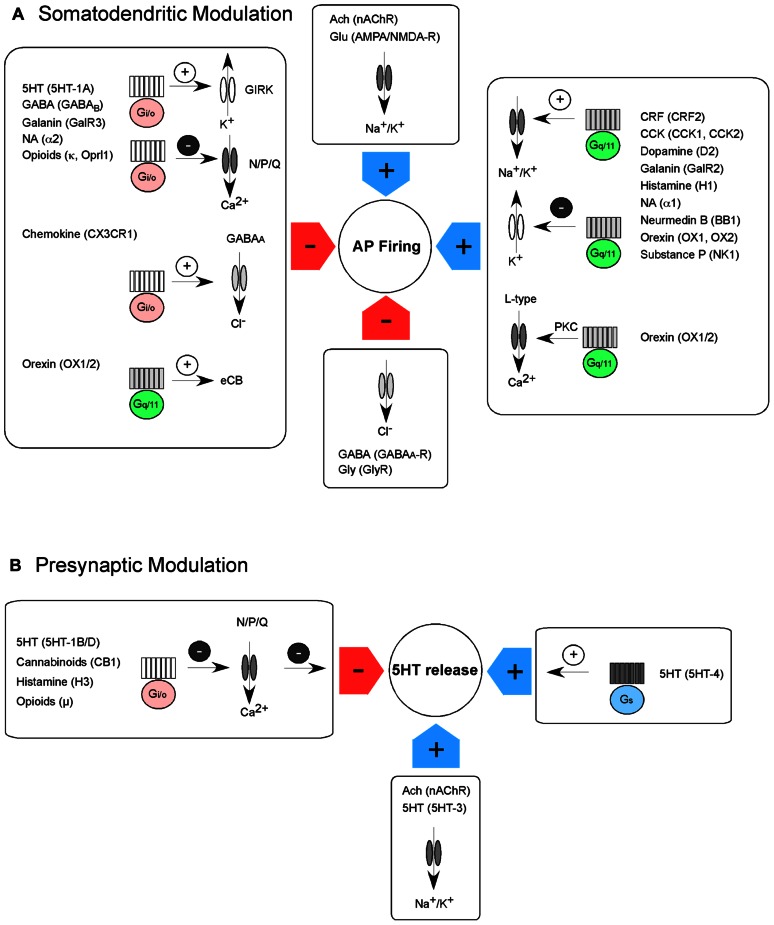
**Intrinsic somatodendritic and presynaptic modulation of action potential (AP) firing and transmitter release in 5-HT neurons**. **(A)** AP firing of 5-HT neurons is modulated by GPCRs and ion channels. AP firing is reduced (red arrows) via activation of GPCRs coupling to the G_i/o_ pathway or activation of inhibitory ligand-gated ion channels. G_i/o_ pathway activation can lead to the activation of GIRK channels, inhibition of voltage-gated Ca^2+^ channels or increase in GABA_A_ receptor currents. In addition, G_q/11_ activation can lead to synthesis of endocannabinoids, which inhibit transmitter release onto 5-HT neurons. AP firing is increased (blue arrows) via activation of GPCRs coupling to the G_q/11_ pathway or activation of excitatory ligand-gated ion channels. G_q/11_ pathway activation can lead to the activation of non-selective cation channels or inhibition of K^+^ conductance. In addition, the activation of L-type Ca^2+^ channels via protein kinase C (PKC) can most likely increase membrane depolarization and transcription. **(B)** 5-HT release of 5-HT neurons is also modulated by GPCRs and ion channels. 5-HT release is reduced (red arrow) via activation of GPCRs coupling to the G_i/o_ pathway. G_i/o_ pathway activation leads to the inhibition of presynaptic Ca^2+^ channels, reduction in Ca^2+^ influx and therefore reduction in transmitter release. 5-HT release is increased (blue arrows) via activation of GPCRs coupling to the G_s_ pathway and via opening of excitatory, ligand-gated ion channels.

The serotonergic system consists of a small number of neurons that are born in the ventral regions of the hindbrain (Deneris and Wyler, 2012). In the adult nervous system, serotonergic neurons [5-HT (5-hydroxytryptamine) neurons] are located in the nine raphe nuclei that are restricted to the basal plate of the midbrain, pons, and medulla (Dahlstrom and Fuxe, 1964). 5-HT neurons located in the rostral raphe nuclei, such as the dorsal raphe nucleus (DRN) and the median raphe nucleus (MRN), give rise to the majority of the serotonergic ascending fibers into the forebrain including cerebral cortex, limbic system, and basal ganglia (Jacobs and Azmitia, 1992). The activity of the serotonergic system is regulated via transmitter release from local interneurons and/or afferents to the raphe nuclei (hetero-regulation), via mechanisms arising from 5-HT neurons themselves (auto-regulation), and potentially via alterations in the extracellular milieu (e.g., increase in CO_2_; Pineyro and Blier, 1999; Richerson, 2004). In this review, we will discuss G protein-coupled receptors (GPCRs) and ion channels located at somatodendritic and presynaptic regions of 5-HT neurons in the DRN and MRN that contribute to the modulation of 5-HT neuronal activity and 5-HT release (**Figure 1**).

The DRN and MRN are the primary nuclei of 5-HT projections to forebrain and provide the neural substrate to communicate between global forebrain and other neuromodulatory systems by sending a wide range of 5-HT projections and receiving a wide variety of afferents (Jacobs and Azmitia, 1992). The DRN is located right beneath the posterior part of cerebella aqueduct and contains about half of all 5-HT neurons in the central nervous system (CNS), which can be further divided into six regions: rostral, caudal, dorsomedial, ventromedial, interfascicular, and lateral parts. The MRN is located at the ventral expansion of the DRN or the midline of the pontine tegmentum where many 5-HT neurons are densely packed in the midline and some 5-HT neurons are scattered in the periphery. Within the DRN and MRN 5-HT neurons project to defined target area in brain (Adell et al., 2002; Lechin et al., 2006). For example, DRN 5-HT neurons innervate the prefrontal cortex, lateral septum, and ventral hippocampus, while MRN 5-HT neurons innervate the temporal cortex, medial septum, and dorsal hippocampus. Afferent projections to the raphe nuclei are diverse and include acetylcholine (ACh) from the laterodorsal tegmental nucleus, dopamine from the substantia nigra and ventral tegmentum area, histamine from tuberomammillary hypothalamic nucleus, noradrenaline (NA) from the locus coeruleus, serotonin itself from the raphe nuclei and several neuropeptides as well as excitatory glutamatergic and inhibitory GABAergic inputs. Glutamatergic inputs come from several nuclei including medial prefrontal cortex and lateral habenula nucleus (Aghajanian and Wang, 1977; Celada et al., 2001; Adell et al., 2002; Bernard and Veh, 2012). Some inputs make direct contacts with 5-HT neurons, while others project onto local GABAergic interneurons that provide feedforward inhibitory input to 5-HT neurons (Sharp et al., 2007). In addition to the local GABAergic interneurons located in the raphe nuclei and in the neighboring periaqueductal gray area, extrinsic GABAergic projections have been suggested (Gervasoni et al., 2000). The responsiveness of 5-HT neurons to each of the inputs differs between DRN and MRN, and also within the subnuclei of the DRN (Adell et al., 2002; Lechin et al., 2006). The differences in responsiveness most likely depend on differences in the strength of the afferent inputs for each of the nuclei and subnuclei and also on the expression and types of the ionotropic and metabotropic receptors in 5-HT neurons. Furthermore, the intrinsic membrane excitability of 5-HT neurons has been reported to differ in the distinct raphe nuclei (Beck et al., 2004; Crawford et al., 2010). Importantly, beyond the anatomical and physiological differences, it has been reported that subpopulation of 5-HT neurons have distinct implications in specific physiological function and behavior (Abrams et al., 2004; Lechin et al., 2006; Hale and Lowry, 2010).

## AUTO-REGULATION

The midbrain 5-HT neurons elicit spontaneous action potentials (APs), with a regular, slow firing pattern (1–5 APs/s; Aghajanian and Vandermaelen, 1982; Vandermaelen and Aghajanian, 1983). 5-HT released from 5-HT neurons act either on the 5-HT neuron itself or on the target circuits. There are several ways how 5-HT neurons may receive 5-HT. First, dendrodendritic synapses releasing 5-HT have been described in raphe nuclei between 5-HT neurons. Second, recurrent axonal collaterals have been suggested to back-propagate to the raphe nucleus itself to release 5-HT. Finally, 5-HT neurons between different raphe nuclei such as DRN and MRN communicate with each other via 5-HT (for review, see Adell et al., 2002; Harsing, 2006; Lechin et al., 2006). Indeed electrical stimulation in DRN slice preparations induces 5-HT_1A_ receptor-mediated slow inhibitory postsynaptic potentials (IPSPs) in 5-HT neurons (Pan et al., 1989; Morikawa et al., 2000), demonstrating 5-HT release in the proximity of 5-HT neurons.

Once 5-HT is released, 5-HT receptors will be activated. Seven subgroups of 5-HT receptors encoding ionotropic as well as metabotropic receptors have been described (5-HT_1_–5-HT_7_), with 15 total variants identified to date (Barnes and Sharp, 1999; Hoyer et al., 2002; Kroeze et al., 2002). The 5-HT GPCRs can be divided into three major subgroups depending on which G protein signaling pathway they activate. 5-HT_1_ receptors couple mainly to the G_i/o_ pathway; 5-HT_4_, 5-HT_5_, 5-HT_6_, and 5-HT_7_ receptors couple to the G_s_ pathway; and 5-HT_2_ receptors activate the G_q/11_ pathway. The 5-HT_3_ receptors are ligand-gated ion channels.

5-HT_1A_, 5-HT_1B_, and 5-HT_1D_ receptors are found on somatodendritic and axonal region of 5-HT neurons (McDevitt and Neumaier, 2011). All three receptors act as negative feedback effectors for 5-HT neuronal firing and 5-HT release (Andrade, 1998). Somatodendritically located 5-HT_1A_ receptors down-regulate the firing rate of 5-HT neurons via activation of G protein-coupled inwardly rectifying potassium channels (GIRK) leading to membrane hyperpolarization, and reduction or complete block of AP firing (Colino and Halliwell, 1987; Sprouse and Aghajanian, 1987; Blier et al., 1989; Hjorth and Sharp, 1991; Penington et al., 1993; Stamford et al., 2000). In addition, 5-HT_1A_ receptors inhibit voltage-gated Ca^2+^ channels of the N- and P/Q-type in 5-HT neurons, as application of a selective 5-HT_1A_ agonist diminishes somatic Ca^2+^ channel currents (Penington and Kelly, 1990; Penington et al., 1992; Bayliss et al., 1997b). The functional consequence of Ca^2+^ channel inhibition is an increase in the firing rate due to reduction in the afterhyperpolarization, which may involve Ca^2+^ activated-K^+^ channel (Bayliss et al., 1997b). The physiological role of the differential effects of 5-HT_1A_ receptors on the AP firing has not been addressed so far, but may involve input specificity due to 5-HT_1A_/GIRK and 5-HT_1A_/Ca^2+^ channel colocalization in specific subcellular domains and/or differences in the regulatory properties of heterogeneous 5-HT neurons within and among different raphe nuclei (Calizo et al., 2011).

The predominant 5-HT receptors at the presynaptic terminal are the 5-HT_1B/1D_ receptors. 5-HT_1B/1D_ receptors have been shown to inhibit 5-HT release from the axonal varicosities as demonstrated with electrophysiological experiments (Sprouse and Aghajanian, 1987; Boeijinga and Boddeke, 1993; Morikawa et al., 2000), probably due to inhibition of Ca^2+^ influx through voltage-gated Ca^2+^ channels such as P/Q-type and N-type Ca^2+^ channel (Kimura et al., 1995; Harvey et al., 1996). The 5-HT_1B_ receptors have been shown to underlie presynaptic autoinhibition of 5-HT release in which 5-HT_1A_-mediated slow IPSPs are reduced by previous released 5-HT activating presynaptic 5-HT_1B_ receptors (Morikawa et al., 2000). In addition, 5-HT_1B_ receptors have been suggested to up-regulate 5-HT reuptake by serotonin transporters (Xie et al., 2008; Hagan et al., 2012) and 5-HT synthesis by itself might be under the control of 5-HT_1B_ (Hjorth et al., 1995). Thus, 5-HT_1B_ autoreceptors may have the ability to control 5-HT release independently from the actual firing rate.

5-HT_1F_ receptor mRNA has also been detected in the raphe nuclei (Bruinvels et al., 1994). Since 5-HT_1F_ receptors have a high affinity for sumatriptan, a 5-HT_1B/1D_ agonist, and the sumatriptan-induced reduction in 5-HT release (monitored by voltammetry in brain slices) could not be blocked by 5-HT_1A/1B/1D_ receptor antagonists, 5-HT_1F_ had been suggested as a possible candidate of a serotonergic autoreceptor in the MRN (Hopwood and Stamford, 2001).

While the expression and function of 5-HT_1_ receptors has been directly demonstrated in 5-HT neurons, involvement of other 5-HT receptors such as 5-HT_2_, 5-HT_3_, and 5-HT_4-7_ is less clear and may differ among species, the developmental stage of the animal and 5-HT neuron subtypes.

5-HT_2_ receptors are functionally expressed in particular on GABAergic interneurons in the DRN, since activation of 5-HT_2A/2C_ receptors increase fast inhibitory postsynaptic current (IPSC) frequency in 5-HT neurons and reduce 5-HT neuronal firing as electrophysiologically measured in brain slices (Liu et al., 2000; Leysen, 2004). 5-HT_2_ receptor mRNA and proteins have been identified in the DRN and embryonic 5-HT neurons (Wright et al., 1995; Clemett et al., 2000; Wylie et al., 2010). Additionally, 5-HT_2_ receptors have been postulated to increase 5-HT_1A_-mediated responses in 5-HT neurons (Kidd et al., 1991). However, a direct modulatory effect of 5-HT_2_ in 5-HT neurons has not been demonstrated.

The 5-HT_3_ receptors have also been suggested to act as presynaptic autoreceptors in serotonergic nerve terminals. Although 5-HT_3_ receptors have been shown to enhance 5-HT release in various brain areas including the raphe nuclei as monitored by [^3^H]5-HT assays (Bagdy et al., 1998), there is no direct immunohistochemical and electrophysiological evidence of the presence of 5-HT_3_ receptors in 5-HT neurons (van Hooft and Vijverberg, 2000).

For the G_s_ protein-coupled 5-HT_4-7_ receptors, mainly indirect evidence exists for an autoregulatory role of these GPCRs in 5-HT neurons.

5-HT_4_ receptors seem to be located somatodendritically and presynaptically. A presynaptic potentiating effect of 5-HT_4_ receptor on glutamate release, which can be counteracted by 5-HT_1A_ receptor-mediated inhibitory action, has been described in hippocampal neurons (Kobayashi et al., 2008). Since neurotransmitter release of various transmitters including 5-HT is modulated by 5-HT_4_ agonists, a presynaptic localization of 5-HT_4_ receptors on 5-HT neurons seems possible (Mengod et al., 2010).

While the function of 5-HT_5_ receptors in the CNS has not been thoroughly studied, two studies suggest a role of 5-HT_5_ receptors for modulating 5-HT neurons. First, 5-HT_5B_ receptor mRNA, a receptor which is expressed in rodents but not in humans, is colocalized with the mRNA of 5-HT transporter in the DRN (Serrats et al., 2004). Second, block of 5-HT_5A_ receptors in the DRN attenuates the 5-carboxamidotryptamine (5-CT; non-selective agonist in particular for 5-HT_1A/1B/1D_ receptors) induced reduction of 5-HT neuronal firing but fail to affect 5-HT release measured using fast cyclic voltammetry *in vitro* (Thomas et al., 2006). The data suggest an autoreceptor modulation of 5-HT neurons via 5-HT_5A_ receptors.

5-HT_6_ and 5-HT_7_ receptor protein and mRNA have been detected in cells in the raphe nuclei including the DRN (Ruat et al., 1993; To et al., 1995; Gustafson et al., 1996; Woolley et al., 2004; see also Gerard et al., 1997; Hamon et al., 1999), but a functional role as autoreceptors for modulating 5-HT neurons could not be demonstrated so far (Bourson et al., 1998; Roberts et al., 2001).

Thus, the auto-regulation of 5-HT neuronal firing is in particular regulated by 5-HT_1A_ receptors via activation of the G_i/o_ pathway and opening K^+^ and closing Ca^2+^ conductance. At the presynaptic terminal, 5-HT_1B/1D_ receptor activation reduces 5-HT release most likely via G_i/o_ protein-mediated inhibition of presynaptic Ca^2+^ channels. In addition, potentiation of 5-HT release by activating 5-HT_4_ receptors via the G_s_ pathway seems possible. Since other 5-HT receptor mRNAs have been detected in 5-HT neurons, other autoregulatory mechanisms may exist in subgroups of 5-HT neurons or during different developmental stages of the serotonergic transmitter system. In particular, animal models for the selective activation of these GPCRs during development will further elucidate the modulatory role of other 5-HT receptors in the auto-regulation of 5-HT neuronal firing and 5-HT release.

## HETERO-REGULATION

5-HT modulates various complex behaviors and therefore the serotonergic transmitter system receives feedback and feedforward information from other brain areas and networks involved in regulating the different behaviors (Adell et al., 2002; Lechin et al., 2006; Sharp et al., 2007). Thus, the hetero-regulation of the 5-HT neurons involves various transmitter systems.

Forty-nine different GPCRs belonging to all four GPCR subfamilies were identified in postmitotic embryonic 5-HT neurons using microarray expression profiling (Wylie et al., 2010). These GPCRs include adrenergic, calcitonin, cannabinoid, GABA, histamine, opioid, and serotonin receptors. For these transmitter systems, a postnatal modulatory role for 5-HT neurons has been described (see below). In addition, other GPCRs such as thrombin, chemokine, prostaglandin E, melanin-concentrating hormone, cadherin, and parathyroid hormone receptors as well as frizzled (FZD) and smoothened (SMO) homolog and the orphan receptors (GPR 19, 56, 85, 98, 125 135, 162, and 173) were also identified but a physiological role for their modulation of 5-HT neurons still needs to be defined and investigated (for a complete list, see Wylie et al., 2010). We will summarize recent findings on the intrinsic hetero-regulation of the serotonergic transmitter system.

### GABA, GLYCINE, AND GLUTAMATE

5-HT neurons within the raphe nuclei receive in particular GABAergic but also glutamatergic input. As expected, the GABAergic input onto 5-HT neurons reduces the neuronal firing, while glutamatergic input increases the firing activity (Pan and Williams, 1989; Levine and Jacobs, 1992; Becquet et al., 1993a,b; for review, see Adell et al., 2002; Harsing, 2006). These effects have been mainly attributed to the expression of ionotropic GABA and glutamate receptors (GluRs) in 5-HT neurons (Tao and Auerbach, 2000; Gartside et al., 2007), which is in agreement with the expression of 2-amino-3-(3-hydroxy-5-methyl-isoxazol-4-yl)propanoic acid (AMPA), *N*-methyl-D-aspartate (NMDA), kainate receptors [GluR 1,2; NMDA-R-2B, kainate receptor 5 (Grik5)] as well as GABA_A_ receptor subunits (GABA_A_ β1–3 and γ2) in embryonic 5-HT neurons (Wylie et al., 2010). Interestingly, the glycine receptor α1 and β subunits have also been identified in human brain and mice embryonic 5-HT neurons (Baer et al., 2003; Wylie et al., 2010). Indeed the DRN receives input from glycinergic fibers (Rampon et al., 1996, 1999) to inhibit 5-HT neuronal firing and 5-HT release (Gallager and Aghajanian, 1976; Wang and Aghajanian, 1977; Becquet et al., 1993a).

The modulation of 5-HT neurons by GABA_B_ receptors has been addressed in various studies. In general, activation of GABA_B_ receptor by selective agonists decreases 5-HT release (Becquet et al., 1993b). The decrease of 5-HT release by GABA_B_ receptors located within 5-HT neurons is most likely mediated via activation of GIRK channels leading to a reduction in AP firing (Innis and Aghajanian, 1987; Williams et al., 1988; Bayliss et al., 1997a; Cornelisse et al., 2007). Within 5-HT neurons, GABA_B_ receptors are located extrasynaptically, suggesting that spillover of GABA during high activity of GABAergic neurons would modulate 5-HT neuronal activity within raphe nuclei (Varga et al., 2002). On the other hand, there is little information about modulatory effects of metabotropic GluRs (mGluRs) in 5-HT neurons so far. Although administration of group II mGluR (mGluR2/3) antagonist has been reported to increase 5-HT neuronal activity, an indirect effect on presynaptic excitatory neurons seems to be involved (Kawashima et al., 2005).

### CORELEASE OF GLUTAMATE OR GABA FROM 5-HT NEURONS

Previous reports have suggested the possibility of glutamate release from 5-HT neurons based on the presence of vesicular glutamate transporter type 3 in a subset of 5-HT neurons (Gras et al., 2002; Amilhon et al., 2010). Using optogenetic techniques, the corelease of glutamate and 5-HT from serotonergic terminals could be demonstrated in a serotonergic projection from the MRN to hippocampal GABAergic interneurons (Varga et al., 2009). The serotonergic fibers make direct synaptic contacts to the GABAergic neurons and exert fast synaptic transmission mediated by ionotropic GluRs and 5-HT_3_ receptors. In addition to the glutamate transporters, GABA and its synthesizing enzyme, glutamic acid decarboxylase (GAD) have also been reported in subsets of 5-HT neurons, suggesting the corelease of GABA and 5-HT (Nanopoulos et al., 1982; Belin et al., 1983; Fu et al., 2010; Hioki et al., 2010; Shikanai et al., 2012). However, vesicular inhibitory amino acid transporter which is necessary for filling synaptic vesicles with GABA was absent in 5-HT/GAD67 positive neurons and their projections, suggesting that GABA may be released by non-vesicular mechanisms such as a reverse transport through GABA transporters (Shikanai et al., 2012). Thus, 5-HT axonal projections have a potential to modulate the neuronal activity in target areas using at least three different transmitters, i.e., 5-HT, glutamate, and GABA. It is intriguing to speculate that the auto-regulation of 5-HT neurons itself might be modulated by corelease of glutamate and GABA.

### ACETYLCHOLINE

The DRN also receives cholinergic input from the laterodorsal tegmental nucleus (Wang et al., 2000). Modulation of 5-HT neurons by ACh mainly involves nicotinic ACh receptors (nAChRs) and can increase 5-HT neuronal firing (Mihailescu et al., 2002) for example, via presynaptic modulation of glutamate release (Garduno et al., 2012) or via opening of nAChR expressed in 5-HT neurons (Galindo-Charles et al., 2008; Chang et al., 2011). Very limited information is available for the expression and function of muscarinic ACh receptors (mAChRs) in 5-HT neurons. mAChR-M_1_ receptors (G_q/11_) might be expressed on serotonergic projections into the hippocampus (Rouse and Levey, 1996) and application of mAChR antagonist, atropine into the DRN enhances antidepressant-like 5-HT_1A_ agonist effects (Haddjeri et al., 2004), which may involve M_2_ (G_i/o_) but not M_1_ (G_q/11_) receptors (Haddjeri et al., 2000). Nevertheless a detailed and direct demonstration of the involvement of mAChRs in 5-HT neurons is essential.

### DOPAMINE

The 5-HT neurons of the DRN reciprocally interact with the dopaminergic mesencephalic transmitter system involving dopamine receptors. D_1_-like receptors (D_1_ and D_5_) couple to the G_s_ pathway, while dopamine D_2_-like receptors (D_2-4_) have been described to couple to the G_i/o_ pathway. In the DRN, D_2_ and D_3_ receptor expression has been detected so far, with very little or no expression of D_1_-like receptors (Bouthenet et al., 1987; Dawson et al., 1988; Cortes et al., 1989; Wamsley et al., 1989; Yokoyama et al., 1994; Suzuki et al., 1998). Dopamine increases 5-HT neuronal firing in DRN *in vivo* and in slice electrophysiological recording, and also increases 5-HT release detected by *in vivo* microdialysis in DRN and other brain areas (Ferre and Artigas, 1993; Ferre et al., 1994; Matsumoto et al., 1996; Mendlin et al., 1998; Haj-Dahmane, 2001; Martin-Ruiz et al., 2001). The modulatory effects on 5-HT neurons have been shown to be mediated by D_1_- and D_2_-like receptors located outside the DRN (Martin-Ruiz et al., 2001) or by direct activation of D_2_ receptors expressed in 5-HT neurons (Haj-Dahmane, 2001). Activation of D_2_-like receptors in 5-HT neurons leads to membrane depolarization, involving activation of G proteins, phospholipase C, and a non-selective cation current, most likely mediated by a transient receptor potential (TRP) channel (Aman et al., 2007). The D_2_-like receptor effects in 5-HT neurons suggest that D_2_-like receptors may activate the G_q/11_ rather than the G_i/o_ signaling pathway. The G_q/11_ protein coupling might be explained by the heterodimerization between D_1_- and D_2_-like receptors (Rashid et al., 2007; Hasbi et al., 2010). It has to be noted that the experiments suggesting the modulation of 5-HT neurons by intrinsic D_2_-like receptors have been performed with relatively high concentrations of quinpirole and sulpiride in *in vitro* preparations. Therefore, the experiments have to be interpreted carefully.

### NORADRENALINE

5-HT neurons in the raphe nuclei receive noradrenergic input in particular from the locus coeruleus (Adell et al., 2002; Lechin et al., 2006). α_1_ and α_2_ adrenergic receptor mRNA and protein have been detected in the DRN and MRN (Unnerstall et al., 1985; Rosin et al., 1993; Scheinin et al., 1994; Talley et al., 1996; Day et al., 1997; Strazielle et al., 1999). α_1_ and α_2_ adrenergic receptors couple to the G_q/11_ and G_i/o_ pathway, respectively. Therefore depending on pathway activation, an increase or decrease in 5-HT neuronal activity and 5-HT release can be postulated if adrenergic receptors are expressed in 5-HT neurons. Early studies revealed that NA causes an increase in 5-HT neuronal firing in DRN. This effect has been suggested to be mediated via activation of α_1_ adrenoceptors (presumably α_1B_ adrenoceptor subtype) located on 5-HT neurons (Vandermaelen and Aghajanian, 1983; Day et al., 1997) and may involve the suppression of a 4-aminopyridine sensitive K^+^ conductance (*I*_A_; Aghajanian, 1985). *In vivo* experiments suggest that α_1_ adrenoceptors are tonically activated by endogenous NA (Adell and Artigas, 1999; Pudovkina et al., 2003). In contrast, 5-HT release detected by voltammetry or [^3^H]5-HT assay in the DRN slice preparation is inhibited by NA, an effect which has been attributed to α_2_ adrenoceptors (involving α_2A_ adrenoceptor subtype) and also perhaps indirectly to α_1_ adrenoceptors (Frankhuijzen et al., 1988; Hopwood and Stamford, 2001). Since α_2_ receptors couple to the G_i/o_ pathway, 5-HT release and 5-HT neuronal firing could be reduced via α_2_ adrenoceptors located at the soma or presynaptic terminal of 5-HT neurons itself (Hopwood and Stamford, 2001), or via inhibition of NA release at noradrenergic synaptic terminals lowering the effective NA concentration for α_1_ adrenoceptors/G_q_ signaling pathway activation. The direct effect of α_2_ adrenoceptors in 5-HT neurons is supported by the fact that embryonic 5-HT neurons express α_2A_ adrenoceptors (Wylie et al., 2010).

### HISTAMINE

There are four histamine receptors (H_1-4_), which couple to different G protein pathways, i.e., H_1_ (G_q/11_), H_2_ (G_s_), H_3_, and H_4_ (G_i/o_). Early studies suggested that histamine reduces the firing of 5-HT neurons in the DRN (Lakoski and Aghajanian, 1983) via H_2_ receptors (Lakoski et al., 1984). Since H_2_ couples to the G_q/11_ pathway, the results suggest that H_2_ receptors are localized on GABAergic terminals. Later findings showed that histamine increased 5-HT neuronal firing in the DRN via activation of H_1_ receptors and the opening of a non-selective cation conductance through G_q/11_ signaling pathways (Barbara et al., 2002; Brown et al., 2002). Expression profiling in mouse embryos suggested the expression of H_3_ receptors in 5-HT neurons, which is consistent with high mRNA levels in the DRN (Lovenberg et al., 1999; Drutel et al., 2001; Pillot et al., 2002). However, the low binding of a H_3_ receptor selective radioligand in the DRN suggests that H_3_ receptors are mainly functional at the presynaptic terminal of 5-HT projections (Pillot et al., 2002). Indeed increasing levels of histamine decrease 5-HT release detected by an *in vivo* electrochemical technique (Hashemi et al., 2011).

### ENDOCANNABINOIDS

The cannabinoid receptor family consists of two subtypes, CB_1_ and CB_2_. Modulation of neuronal activity is mainly exerted via CB_1_, which couples to the G_i/o_ protein (Howlett et al., 1986) and probably also to the G_s_ pathway (Glass and Felder, 1997). CB_1_ receptors are localized in particular at presynaptic terminals, where they inhibit presynaptic Ca^2+^ influx and reduce transmitter release via endocannabinoid (eCB)-mediated retrograde signaling (Maejima et al., 2001). CB_1_ receptors are expressed in serotonergic fibers (Haring et al., 2007; Ferreira et al., 2012) and their mRNA is found early in development (Wylie et al., 2010). 5-HT release in projection areas from the DRN is reduced by activation of CB_1_ as monitored by microdialysis (Egashira et al., 2002) and [^3^H]5-HT assay (Nakazi et al., 2000; Egashira et al., 2002), and increased by inhibition of CB_1_ *in vivo* and *in vitro* (Darmani et al., 2003; Tzavara et al., 2003; Aso et al., 2009). Studies in CB_1_ knock-out animals and chronic activation of CB_1_ receptors *in vivo* suggest that CB_1_ may regulate the function and expression of 5-HT_1A_ receptors (Aso et al., 2009; Moranta et al., 2009; Zavitsanou et al., 2010). Interestingly, 5-HT neurons itself synthesize eCBs in an activity-dependent manner (Haj-Dahmane and Shen, 2009, 2011). eCB release from 5-HT neurons can be induced by orexin-B leading to the activation of orexin (OX) receptors via the G_q/11_ pathway (Liu et al., 2002b; Haj-Dahmane and Shen, 2005). It has been therefore speculated that the activity-dependent activation of the G_q/11_ pathway in general may lead to the production of eCBs in 5-HT neurons (Haj-Dahmane and Shen, 2011). Within the DRN eCBs mainly act on glutamatergic terminals and probably also on GABAergic terminals (Liu et al., 2002b; Haj-Dahmane and Shen, 2009; Mendiguren and Pineda, 2009; Tao and Ma, 2012), leading to a reduction in glutamate and GABA release onto 5-HT neurons and therefore changes the activity of the 5-HT neurons itself.

### FRIZZLED RECEPTORS

Four frizzled receptors (FZD1–3 and SMO) have been detected in postmitotic embryonic 5-HT neurons (Wylie et al., 2010). These receptors mainly couple to the Wnt signaling cascade and are most likely involved in the development and maturation of 5-HT neurons (Simon et al., 2005; Song et al., 2012). However, the role of frizzled receptors in 5-HT neurons remains to be determined.

### NEUROPEPTIDES

Dr. Hökfelt’s laboratory demonstrated that various peptide transmitters are expressed in the DRN with species-specific differences between mice and rats (Fu et al., 2010). The identified neuropeptides are cholecystokinin (CCK), calcitonin gene-related peptide (CGRP), vasoactive intestinal peptide (VIP), somatostatin, substance P, dynorphin, neurotensin, thyrotropin-releasing hormone (TRH), enkephalin, galanin, neuropeptide Y (NPY), and corticotropin-releasing factor (CRF). Among these, various peptide receptors have been identified and functionally described in 5-HT neurons.

### BOMBESIN

To date three types of bombesin receptors have been described, i.e., the neuromedin B (NMB) receptor (BB_1_ receptor), the gastrin-releasing peptide (GRP) receptor (BB_2_ receptor) and the bombesin receptor subtype 3 (BRS-3 or BB_3_ receptor; Jensen et al., 2008). The NMB receptors are functionally expressed in 5-HT neurons in the DRN (Pinnock et al., 1994; Woodruff et al., 1996). BB_1_ receptor activation leads to an increase in 5-HT neuronal firing via suppression of K^+^ current, involving most likely the activation of the G_q/11_ pathway (Benya et al., 1992; Woodruff et al., 1996) and as a consequence, an increase in 5-HT release to projection site such as the hippocampus, which is monitored by *in vivo* microdialysis (Merali et al., 2006).

### CALCITONIN

Calcitonin receptor (CalcR) mRNA has been localized in 5-HT neurons in the DRN (Nakamoto et al., 2000), which is in agreement with the expression profiling studies of postmitotic embryonic 5-HT neurons (Wylie et al., 2010). The CalcR couples to the G_s_ pathway (Hay et al., 2005) and G_q/11_ pathway (Offermanns et al., 1996). Since high levels of amylin binding sites are detected in the DRN (Sexton et al., 1994) and mRNA for CGRP has been localized in 5-HT positive axon terminals in monkeys (Arvidsson et al., 1990), it is most likely that CalcR assemble with receptor activity-modifying proteins (RAMPs) in 5-HT neurons to respond to the various peptide transmitters (i.e., CGRP, adrenomedullin, and amylin; Tilakaratne et al., 2000). A direct function of CalcR in 5-HT neurons has not yet been demonstrated. However, injection of CGRP into rats induces anxiety-like behaviors and increases c-Fos expression in the DRN (Sink et al., 2011).

### CHEMOKINE RECEPTORS

Two subtypes of the 18 identified chemokine receptors have been detected in microarray analyses from embryonic 5-HT neurons, i.e., Duffy antigen/chemokine (C-X-C motif) receptor 4 (CXCR4; Wylie et al., 2010). CXCR4 is expressed in the majority of 5-HT neuron outer membranes in the DRN (Heinisch and Kirby, 2010). So far only an indirect action of CXCR4 for modulation of 5-HT neuron has been demonstrated, since application of the CXCR4 ligands and antagonists modulate GABA and glutamate release onto 5-HT neurons (Heinisch and Kirby, 2010), which is in agreement with the described G_i/o_ protein-mediated inhibition of Ca^2+^ channels via CXCR4 (Oh et al., 2002). In addition, CX_3_CR1 is also expressed in 5-HT neurons in the DRN and MRN (Heinisch and Kirby, 2009). Here the CX_3_CR1 specific ligand, fractalkine/CX_3_CL1 increased evoked IPSC amplitude on 5-HT neurons (Heinisch and Kirby, 2009), an effect which is most likely mediated postsynaptically and not presynaptically. The effect is surprising, since CX_3_CR1 has been described to couple to the G_i/o_ pathway (Oh et al., 2002), which would inhibit synaptic transmitter release and induce paired-pulse facilitation (PPF) if activated on GABAergic terminals. Therefore, the CX_3_CL1 could increase/modulate GABA_A_ receptor trafficking and GABA_A_ receptor currents in 5-HT neurons via activation of CX3CR1. A signaling function for Duffy antigen remains to be determined.

### CHOLECYSTOKININ

The expression of CCK receptors in 5-HT neurons in the DRN has also been suggested. Application of CCK increases 5-HT neuronal firing, which is blocked by the CCK_1_ antagonist L-364,718 (Boden et al., 1991). In addition, 5-HT release measured as outflow of [^3^H]5-HT in cortical slices is increased by CCK-4, which involves CCK_2_ receptors (Siniscalchi et al., 2001). Both CCK_1_ and CCK_2_ receptors mainly couple to the G_q/11_ and G_s_ pathway (de Weerth et al., 1993; Lee et al., 1993; Ulrich et al., 1993).

### CORTICOTROPIN-RELEASING FACTOR

Two CRF (CRF_1_ and CRF_2_) receptor subtypes have been described in brain and both seem to be localized in GABAergic neurons in the DRN as well as in 5-HT and non-5-HT neurons with differential subcellular localizations (for review, see Valentino et al., 2010). CRF_1_ and CRF_2_ couple to the G_s_ pathway (Chang et al., 1993; Perrin et al., 1993; Vita et al., 1993; Lovenberg et al., 1995; Liaw et al., 1996) and also the G_q/11_ pathway in heterologous expression systems (Dautzenberg et al., 2004), leading to the assumption that stimulation of CRF_1_ or CRF_2_ will increase neuronal firing. Indeed, activation of CRF_1_ on GABAergic neurons increases GABA release onto 5-HT neurons, while activation of CRF_2_ elicits an inward current in 5-HT neurons (Kirby et al., 2008). Based on the differential localization of the CRF receptors within the DRN and its receptor type-specific action on 5-HT neurons, it has been suggested that at low concentrations of CRF, 5-HT neuronal activity is decreased, while at high concentrations, 5-HT neuronal activity is increased (Kirby et al., 2008; Valentino et al., 2010).

### GALANIN

The neuropeptide galanin activates three types of galanin receptors (GalR_1-3_). GalR_1_ and GalR_2_ are highly expressed in the DRN (Melander et al., 1988; Larm et al., 2003; Lu et al., 2005; Sharkey et al., 2008). Moreover, GalR_1_ expression has also been described in 5-HT neurons from rats but not in mice (Xu et al., 1998; Larm et al., 2003). GalR activation in the DRN causes a K^+^ conductance-mediated hyperpolarization in rat brain slices (Xu et al., 1998), most likely via GalR3-mediated G_i/o_ activation of GIRK channels (Swanson et al., 2005). These effects are in agreement with *in vivo* microdialysis studies showing that injection of galanin into the DRN reduces 5-HT release via GalR activation in the hippocampus (Kehr et al., 2002). In contrast to the inhibitory action of galanin on 5-HT neuronal activity, a reduction in inhibitory input onto 5-HT neurons has also been described (Sharkey et al., 2008). Here, the pan GalR_1-3_ agonist reduced GABA-mediated fast synaptic transmission accompanied by increase of PPF, suggesting that GalRs are expressed on GABAergic terminals and inhibit presynaptic Ca^2+^ channels via the G_i/o_ pathway. On the other hand, GalR_2_ agonist, galanin (2–11) reduced IPSP amplitude but did not cause PPF, suggesting a postsynaptic action (Branchek et al., 2000; Sharkey et al., 2008). Additionally, galanin (2–11) was demonstrated to increase 5-HT release in hippocampal tissue by immunofluorescence and high-performance liquid chromatography (HPLC) measurement (Mazarati et al., 2005). Therefore GalR_2_ receptors may activate 5-HT neurons via reduction in GABAergic input onto 5-HT neurons and/or via G_q/11_-mediated increase in 5-HT neuronal firing. Galanin also modulates 5-HT_1A_ autoreceptor responses *in vivo*. A possible mechanism of this modulation could be the heterodimerization of GalR with 5-HT_1A_ which has been observed in heterologous expression systems (for review, see Kuteeva et al., 2008; Borroto-Escuela et al., 2010).

### HYPOCRETIN–OREXIN

The two hypocretin/orexin (OX1 and OX2) receptors are expressed in tryptophan hydroxylase-positive neurons in the DRN (Brown et al., 2002). Their intracellular signaling targets are rather complex involving activation of G_i/o_, G_q/11_, G_s_, and other G proteins (Scammell and Winrow, 2010). Orexin positive fibers project onto GABAergic as well as 5-HT neurons in the DRN (Peyron et al., 1998). Application of the neuropeptides orexin-A and orexin-B causes a Na^+^/K^+^ non-selective cation current in 5-HT neurons (Brown et al., 2002; Liu et al., 2002b; Kohlmeier et al., 2008), suggesting that activation of OX1 and OX2 leads to the increase of 5-HT neuronal firing. The neuropeptides also induce GABA release onto 5-HT neurons at higher peptide concentrations (Liu et al., 2002b). In addition, orexin increases the somatic L-type Ca^2+^ current in 5-HT neurons in a protein kinase C-dependent manner (Kohlmeier et al., 2008). It has therefore been suggested that modulation of Ca^2+^ transients by orexin may be involved in the transcriptional regulation of long-term processes (Kohlmeier et al., 2008).

### OPIOIDS

Raphe nuclei receive dynorphinergic, enkephalinergic, and β-endophinergic innervation (Adell et al., 2002). These transmitters activate μ, κ and δ opioid receptors, which primarily couple to the G_i/o_ pathway. Injection of morphine into the DRN causes an increase in 5-HT release detected in forebrain microdialysis (Tao and Auerbach, 1994). The increase in 5-HT release is most likely mediated via G_i/o_ protein-mediated inhibition of GABAergic interneurons in the DRN, involving μ opioid receptors located on GABAergic neurons (Jolas and Aghajanian, 1997). The modulation of 5-HT release in the DRN by κ and δ opioid receptors has also been described (Tao and Auerbach, 2002). Activation of δ receptors increased, while activation of κ receptors decreased 5-HT release measured with *in vivo* microdialysis. The κ receptors effects do not involve the modulation of GABAergic or glutamatergic inputs in the DRN (Tao and Auerbach, 2002), suggesting that κ receptors are expressed in 5-HT neurons. Likewise, opioid receptor-like1 (Oprl1) are most likely located and expressed in 5-HT neurons as follows. Oprl1 or nociceptin (NOP) receptors belong to the opioid receptor family but are activated by NOP (orphanin FQ), a neuropeptide derived from prepronociceptin protein. High levels of NOP receptor binding sites have been detected in the DRN (Florin et al., 2000) and Oprl1 receptor expression could be detected in embryonic 5-HT neurons (Wylie et al., 2010). NOP/orphanin FQ inhibits 5-HT release in the DRN via Oprl1 (Tao et al., 2007), suggesting a functional role of Oprl1 in 5-HT neurons early in development and in the adult brain.

### SUBSTANCE P

Substance P belongs to the tachykinin family and has a high affinity for the three different neurokinin receptors (NK_1-3_), in particular to NK_1_ (Hokfelt et al., 2001). These GPCRs couple mainly to the G_q/11_ pathway (Stratowa et al., 1995), but G_s_ pathway activation has also been reported for NK_1_ in cell culture systems (Martini et al., 2002). Various histological studies have revealed extensive expression of NK_1_ receptors in the DRN (Maeno et al., 1993; Saffroy et al., 1994; Vigna et al., 1994; Charara and Parent, 1998; Sergeyev et al., 1999; Froger et al., 2001). Most studies suggest that NK_1_ receptors are not localized on 5-HT neurons (Froger et al., 2001; Santarelli et al., 2001), while others revealed NK_1_ receptor expression in a subpopulation of 5-HT neurons (Santarelli et al., 2001; Lacoste et al., 2006, 2009). Interestingly, NK_1_ receptors are found in the cytoplasm of the 5-HT neurons and in dendritic membranes of GABAergic neurons. After administration of NK1 antagonist or deafferentation of substance P releasing projections, the density of membrane bound NK_1_ receptors is increased in the somatodendritic region of 5-HT neurons, suggesting that membrane trafficking of NK_1_ receptors may be regulated by Substance P input. This mechanism may contribute to the modulation of 5-HT neuronal firing under certain physiological conditions (Lacoste et al., 2009). In addition, controversial results exist for the effect of NK_1_ on 5-HT release and firing of 5-HT neurons. Inhibition of NK_1_ in the DRN using antagonists or knock-out strategies leads to an increase in firing activity of 5-HT neurons *in vivo* (Haddjeri and Blier, 2001; Santarelli et al., 2001). In contrast, activation of NK_1_ and NK_3_ increases spontaneous excitatory postsynaptic currents (EPSCs) in DRN 5-HT neurons resulting in an increased firing of the 5-HT neurons as observed in brain slice recording (Liu et al., 2002a). These effects could be blocked by NK_1_ and NK_3_ antagonists (Liu et al., 2002a). Also, activation of NK_1_ via intra-raphe injection of substance P in the DRN increases 5-HT release within the DRN, but decreases 5-HT release in frontal cortex as measured with *in vivo* microdialysis (Guiard et al., 2007). These effects and also the described increase in 5-HT firing in NK_1_ knock-out mice involve changes in 5-HT_1A_ autoreceptor levels, suggesting at least a functional coupling between NK_1_ and 5-HT_1A_ receptors. Further investigations to verify these interactions in 5-HT neurons are required.

In summary, the various heteroreceptors integrate incoming information via two main pathways, i.e., G_i/o_ and G_q/11_ leading to inhibition or activation of 5-HT neuronal firing and 5-HT release, respectively. Besides the “classical” G_i/o_ protein-mediated, membrane-delimited modulation of GIRK and presynaptic Ca^2+^ channels, other ion channel targets have been identified in 5-HT neurons. For example, two-pore-domain K^+^ channels [TWIK-related acid-sensitive K-1 (TASK-1) and TASK-3] have been described in dorsal and caudal raphe 5-HT neurons (Washburn et al., 2002). TASK channels are inhibited by GPCRs coupling to the G_q/11_ pathway most likely in a membrane-delimited manner involving the direct binding of Gαq subunits (Chen et al., 2006). The existence of voltage-sensitive but not ATP-dependent K^+^ channels in DRN neurons including 5-HT neurons have been proposed based on drug application studies (Harsing, 2006). In addition, TRP channels have been described to be modulated by D_2_-like receptors (Aman et al., 2007). According to the microarray expression profiling studies, various ion channel targets of GPCRs are expressed in embryonic 5-HT neurons including TRP (Trpm4 and Trpm7), two-pore channels (TPCN1), cyclic nucleotide gate channels (Hcn3), and KCNQ (Kcnq2; Wylie et al., 2010). Therefore, more detailed studies have to be performed to determine the role of other ion channel targets and in particular long-term effects of GPCR modulation for the serotonergic system.

## INTEGRATION AND SIGNAL PROCESSING OF MODULATORY INFORMATION BY SEROTONERGIC NEURONS: WHY SO MANY GPCRs?

The serotonergic transmitter system modulates many physiological functions such as mood, sexual behavior, feeding, sleep/wake cycle, memory, cognition, blood pressure regulation, breathing, and reproductive success (Mooney et al., 1998; Abrams et al., 2004; Lechin et al., 2006; Lerch-Haner et al., 2008; McDevitt and Neumaier, 2011). Because of the complexity and variety of the different behaviors modulated by serotonin, it is expected that modulatory signals from other brain areas including sensory information is integrated by GPCR signals in nuclei containing 5-HT neurons using a high diversity of GPCRs. While GABA and glutamatergic input into the raphe nuclei will adjust 5-HT neurons to the current inhibitory/excitatory state of the brain, other transmitter systems will inform 5-HT neurons more directly about the serotonin-associated behavior. For example, dopamine is involved in reward-driven learning; ACh modulates arousal and reward; NA and CRF are involved in stress responses; histamine is involved in sleep regulation and sexual function; bombesin and CCK regulate eating behavior; eCBs modulate memory, appetite, stress, social behavior, anxiety, and sleep; galanin has been implicated in the regulation of sleep–wake cycle, cognition, emotion, and blood pressure; hypocretin–orexin modulate arousal, wakefulness, and appetite; and opioids and substance P are involved in pain perception and mood (White and Rumbold, 1988; Woodruff et al., 1996; Greenough et al., 1998; Bear et al., 2001; Merali et al., 2006; Monti, 2010; Haj-Dahmane and Shen, 2011). Since all different behavioral responses can be integrated in nuclei containing 5-HT neurons, regulation of serotonin release will affect similar behaviors as stated above. Thus there is a tight interaction and signaling exchange between the different transmitter systems to precisely modulate behavioral output. Consequently, long-term changes in serotonin release can involve changes in the auto-regulation involving 5-HT receptor or hetero-regulation involving the above mentioned GPCRs and can cause neuropsychiatric disorders, most notably depression, anxiety, schizophrenia, and dementia (Lucki, 1998; Davidson et al., 2000; Mann et al., 2001; Nelson and Chiavegatto, 2001). The modulation of the different behaviors is even more complex since other GPCRs, such as orphan GPCRs, with so far unknown function are also expressed in 5-HT neurons. Therefore, new strategies and techniques have to be applied and developed to understand complex behaviors related to the serotonergic system.

## NEW APPROACHES TO CONTROL AND UNDERSTAND SEROTONERGIC G PROTEIN-COUPLED RECEPTOR SIGNALING PATHWAYS

Recently, several new approaches to manipulate the activity of 5-HT neurons in a cell type-specific manner have been developed. Various promoter/enhancer sequences have been isolated and characterized, which allow for the expression of proteins of choice within at least subsets of 5-HT neurons. These DNA sequences include the promoter or enhancer sequences of Pet-1/Fev transcription factor, the serotonin transporter (SLC6A4), and the tryptophan hydroxylase 2 (TPH2; Scott et al., 2005). Using these different promoter/enhancer sequences, different mouse lines and virus approaches have been developed to activate and/or silence/delete reporter genes such as green fluorescent protein (GFP) or tdTomato, Cre or Flip recombinases, tetracycline inducible systems, tetanus toxin light chain, and genes of interest (Scott et al., 2005; Kim et al., 2009; Madisen et al., 2010, 2012; Richardson-Jones et al., 2010; Zhao et al., 2011). Using the tetracycline inducible system, for example, the genetic ablation of 5-HT_1A_ from the majority of 5-HT neurons could be achieved (Richardson-Jones et al., 2010, 2011).

For the investigation of the modulation and function of neuronal circuits in general and for the serotonergic system in particular, chemical and optogenetic techniques have been developed in recent years (Herlitze and Landmesser, 2007; Masseck et al., 2010). For control of neuronal activity in various neuronal circuits including the 5-HT system, the light-gated non-selective cation channel, ChR2 has been used and allows for the dissection of 5-HT-mediated behavioral effects in different raphe nuclei (Li et al., 2005; Varga et al., 2009; Zhao et al., 2011; Madisen et al., 2012; see also Kim et al., 2009). For the investigation of GPCR signals, various chemically and light-activated GPCRs have been developed (Masseck et al., 2010). For example vertebrate rhodopsin (vRh) has been used to regulate G_i/o_ signaling pathways in neurons by light (Li et al., 2005). Exogenously expressed vRh inhibits neuronal firing and neurotransmitter release *in vitro* and *in vivo* most likely via activation of GIRK and inhibition of presynaptic Ca^2+^ channels (Li et al., 2005; Oh et al., 2010; Gutierrez et al., 2011). Since vRh belongs to class A or rhodopsin like group of GPCRs, like serotonergic autoreceptors 5-HT_1A/1B/1D_, the idea arose to generate light-activated chimeric receptors which couple to intracellular signaling pathways in 5-HT_1_ receptor domains. The feasibility of receptor domain swapping has been demonstrated by Dr. Khorana’s group (Kim et al., 2005). They replaced the intracellular loops of vRh with that of the β_2_-adrenergic receptor and turned vRh into a light-activated G_s_ protein-coupled receptor, Opto-β_2_AR. The approach however was not suitable for the exchange of vRh intracellular peptide loops by the 5-HT_1A_ intracellular receptor domains, since this chimeric receptor revealed altered activation and deactivation kinetics in respect to GIRK channel modulation (Oh et al., 2010). However, it could be shown that the C-terminus (CT) of the 5-HT_1A_ receptor was sufficient to target vRh into expression domains of 5-HT_1A_ receptor and functionally substitute for G_i/o_ pathway activation. The CTs of GPCRs contain peptide signal domains for subcellular targeting and G protein interaction. The CT of 5-HT_1A_ had been shown to be necessary for trafficking of the receptor to dendritic domains via interaction with trafficking protein Yif1B (Carrel et al., 2008). Fusion of the 5-HT_1A_ receptor CT onto vRh was therefore sufficient to target the chimeric construct Rh-CT_5-HT1A_ into somatodendritic regions of hippocampal neurons and 5-HT neurons in the DRN and exclude the expression of the chimeric receptor from the axons. The Rh-CT_5-HT1A_ receptors were able to functionally substitute for intracellular 5-HT_1A_ signals in DRN neurons of 5-HT_1A_ knock-out mice, i.e., light illumination induced K^+^ conductance (most likely GIRK) reduced the firing rate of spontaneously active 5-HT neurons. Thus, the adjustment of for example light-activated or chemically activated GPCRs and their coupling and anchoring to and in intracellular signaling domains will allow for the dissection of multiple GPCR pathways within the serotonergic system and their interaction *in vitro* and *in vivo*.

## Conflict of Interest Statement

The authors declare that the research was conducted in the absence of any commercial or financial relationships that could be construed as a potential conflict of interest.
